# The Role of Change in the Relationships Between Leader-Member Exchange/Coworker Exchange and Newcomer Performance: A Latent Growth Modeling Approach

**DOI:** 10.3389/fpsyg.2021.600712

**Published:** 2021-05-13

**Authors:** Jing Liu, Allan Lee, Xueling Li, Ci-Rong Li

**Affiliations:** ^1^School of Management, Jilin University, Changchun, China; ^2^Exeter Business School, University of Exeter, Exeter, United Kingdom

**Keywords:** leader-member exchange relationship, coworker exchange relationship, newcomer performance, psychological entitlement, conscientiousness

## Abstract

This study examines whether and how the qualities of newcomers’ interpersonal relationships [i.e., leader-member exchange (LMX) and coworker exchange (CWX)] relate to their initial performance and how changes in the qualities of these relationships relate to the changes in performance. To test a latent growth model, we collected data from 230 newcomers at six time points over a 6-week period. The results showed that LMX quality is positively related to initial newcomer performance; however, changes in LMX quality are not statistically significantly related to changes in newcomer performance. In contrast, an increase in CWX quality is positively related to newcomer performance improvement, but the initial quality of CWX does not predict newcomer performance. Furthermore, newcomers’ psychological entitlement moderates the relationship between LMX quality and newcomer performance; newcomers’ conscientiousness moderates the relationship between increases in CWX quality and improvements in newcomer performance. The findings increase our understanding of the newcomer exchange relationship-performance link over time and suggest that future newcomer socialization research explore the initial level of and the changes in these relationships simultaneously.

## Introduction

Organizational socialization, which is at its most intense in the initial weeks and months after entry (Van Maane and Schein, 1979), is the process by which new employees acquire the knowledge and skills needed to perform their role and function effectively in the new environment (e.g., Allen et al., 2017). The early experiences of new employees are pivotal for determining their subsequent job attitudes and behaviors and whether they will remain with the organization (e.g., Bauer et al., 2007; Saks et al., 2007). Research highlights that employee socialization is influenced, to a large extent, by the relationships that employees develop in these early stages of organizational entry (e.g., Fang et al., 2011). In this regard, previous research has identified “organizational insiders,” such as leaders and coworkers, as key socializing agents (e.g., Ostroff and Kozlowski, 1992; Nifadkar et al., 2012). For instance, leaders, as supporters, provide knowledge and feedback to help the newcomers master work tasks and clarify their role identity (Jokisaari, 2013); coworkers as collaborators, communicate, coordinate, and cooperate to accomplish the tasks (Kelly and McGrath, 1985; Gersick, 1988, 1989; Ganegoda and Bordia, 2019). For newcomers, developing better interpersonal relationships allows better adjustment to life in a new organization (Saks and Gruman, 2012). Among other traditional outcomes (e.g., job satisfaction and commitment), we propose that newcomer performance could be the objective form of newcomer adjustment, as higher performance indicates a better adjustment to this job role.

Building on the argument that newcomers are socialized largely through interpersonal interactions with organizational insiders (Ashforth et al., 2007; Kammeyer-Mueller et al., 2013), previous research provides empirical support for a positive association between newcomer relationships with such insiders and their adjustment and performance (e.g., Liden et al., 1993; Major et al., 1995; Chen and Eldridge, 2011; Sluss and Thompson, 2012). However, to date, most of this research has focused on the relationship between newcomers and their supervisors, assuming that supervisors are the most influential source of localized socialization for newcomers (Ashforth et al., 2007; Sluss and Thompson, 2012). In contrast, there is a relative paucity of research exploring the role that coworker relationships play in newcomer socialization. Thus, while it is important to consider how vertical relationships (e.g., with one’s supervisor) can impact newcomer adjustment, the salience of that topic in the current literature somewhat diminishes the importance of horizontal relationships that exist among coworkers and work teams (Banks et al., 2014).

Drawing upon social exchange theory (SET; Blau, 1964; Coyle-Shapiro and Conway, 2004), we suggest that newcomers’ relationships with their leader and coworkers will have important consequences for their performance and that employee conscientiousness and psychological entitlement will moderate these effects. Our research contributes to the newcomer socialization literature in three main ways. First, we explore the relative effects of LMX and CWX in influencing newcomer performance. While previous research has demonstrated the effects of either LMX or CWX on newcomer adjustment, the current research explores the concurrent effects of both horizontal and vertical exchanges. For organizations, knowing where to focus their resources during newcomers’ organizational entry is crucial. Our research contributes by extending the understanding of the relative effects of LMX and CWX on newcomer performance.

Second, our study provides an investigation of the change patterns of LMX and CWX during newcomers’ early socialization process, and thus contributes to revealing the dynamic process of newcomer socialization and performance. Organizational socialization, by its very nature, is a dynamic process whereby newcomers adjust to and integrate within the organization (Allen et al., 2017). As far back as 1986, Fisher (1986, p. 103) noted that “socialization is a dynamic process in which individuals and organizations change over time. Many studies have failed to handle the time dimension appropriately.” Despite this call being made over 30 years ago, a recent review of the organizational socialization literature (Allen et al., 2017) highlights that the failure to capture the dynamics of organizational socialization continues to be a major limitation of the literature. Although some research has considered both LMX and CWX as temporal frames of reference (Nahrgang et al., 2009; Guarana and Barnes, 2017), few studies have examined the interpersonal relationships that impact newcomer adjustment not only in terms of levels but also changes. Indeed, the level and the change of the qualities of LMX and CWX may often produce different forms of variance, which may capture unique information that is not accounted for in a single perspective (Ployhart and Vandenberg, 2010). Therefore, the present research adds to the literature by examining the longitudinal effects of LMX and CWX on newcomer performance during organizational socialization. Using latent growth modeling, we address calls to better understand the dynamics of both organizational socialization (e.g., Allen et al., 2017) and LMX (e.g., Castillo and Trinh, 2018). This research design allows us to examine how LMX/CWX predicts newcomer performance and trajectories of change in these constructs.

The third contribution made by this current research is to uncover the role that employee personality plays in shaping the effects of LMX and CWX on newcomer performance. To date, there is a lack of understanding around whether contextual factors exist that may help explain when LMX and/or CWX is more likely to influence newcomer performance. Building on social exchange theory and previous research, we argue that employees’ level of psychological entitlement and conscientiousness will moderate the effects of LMX and CWX. Thus, whereas previous research has assumed that a high-quality relationship with one’s leader and coworkers will be beneficial for newcomer adjustment (e.g., Sluss and Thompson, 2012), we argue that this relationship will be influenced by followers’ personality.

## Theoretical Development

The early period of organizational socialization among newcomers is contingent on the interaction between the newcomers and organizational insiders (i.e., supervisors and coworkers). Such interactions and exchanges shape newcomers’ knowledge and understanding of the job, role, group environment, and organizational culture (e.g., Slaughter and Zickar, 2006; Li et al., 2011). Past research has demonstrated that during the early stage of socialization, newcomers feel uncertainty about their surroundings (Ellis et al., 2015), which can be reduced by the positive interpersonal exchange relationships (i.e., LMX and CWX; Major et al., 1995). Several studies have revealed that the quality of LMX is positively associated with favorable outcomes during socialization (Dulebohn et al., 2012; Martin et al., 2016). Although more recent studies have noted the significant role coworkers play in the organizational life (Baker and Omilion-Hodges, 2013; Omilion-Hodges et al., 2016; Omilion-Hodges and Ackerman, 2018), no study has yet explored the relative effect of LMX and CWX on newcomer performance. In addition, past studies have typically focused on interpersonal relationships cross-sectionally, neglecting the change in those variables and whether or how those changes influence the outcomes. The research of Nahrgang et al. (2009) assessed the quality of LMX over repeated timepoints and concluded that LMX quality changed considerably over a period of 8 weeks; however, it did not consider how newcomer performance will fluctuate with LMX quality. Although newcomer performance fluctuates during the early period of socialization (Chan and Schmitt, 2000), far less research has examined the dynamic nature of newcomer socialization and the specific impact of leaders and coworkers on newcomer performance. Therefore, our study examines whether and when the quality of interpersonal exchange relationships that a newcomer develops with leaders (LMX) and coworkers (CWX) simultaneously and relatively influence newcomer adjustment in a parallel fashion.

Scholars have increasingly adopted a SET (Blau, 1964) lens to understand how interpersonal exchange relationships in work settings influence workplace behavior (e.g., Lee et al., 2019a). SET is built on the principle of reciprocity (Gouldner, 1960) and posits that individuals will feel compelled to reciprocate a positive exchange from another individual. Based on this, Lyons and Scott (2012) investigated “homeomorphic reciprocity,” in which one party initiates a favorable interaction (e.g., being helpful), and the other party also behaves favorably (e.g., being helpful), and similarly, unfavorable behaviors (e.g., being harmful) also begets behave unfavorable behavior (e.g., being harmful). The concept emphasizes the matching exchange between the two parties (Cropanzano et al., 2017). As such, the better the perceived quality of the LMX/CWX relationship, the more determined individuals are to invest in the social exchange relationship. As one initiates the exchange in kind, the other party reciprocates, and the relationship deepens, generating a self-reinforcing cycle (Cropanzano and Mitchell, 2005; Cropanzano et al., 2017). Accordingly, when a follower receives favorable treatment from their leader or coworker, it should create feelings of obligation to repay the other party by exerting increased effort as a means of reciprocation. This effort, in turn, should enhance followers’ task performance. These arguments are well-supported by growing empirical work highlighting the positive association between LMX quality and follower performance (e.g., Dulebohn et al., 2012; Martin et al., 2016).

### LMX and Newcomer Adjustment

Newcomers lack organizational experience and are required to simultaneously master their tasks and comprehend their role responsibility in a short period of time (Saks and Gruman, 2012). Leaders, as the formal authority in the organization, serve as a main channel for newcomers to learn about the job and the organization (e.g., Major et al., 1995; Jokisaari, 2013). More importantly, leaders can be flexible about the responsibilities they delegate and the work they assign. Compared to newcomers with low-quality of LMX, their counterparts with high-quality LMX can access more vertical resources (Tse and Dasborough, 2008; Fang et al., 2017). For example, when employees first enter a new organization, they normally feel uncertain about work and seek more referent information (e.g., about their job responsibilities), which may be best provided by their immediate supervisors who determine their job description. High-quality exchanges between leaders and newcomers facilitate the enquiry in this vertical relationship and clarify the role’s responsibilities. Moreover, such frequent communications about work can also strengthen the newcomers’ communicative relationships (Omilion-Hodges and Ackerman, 2018). Studies have also shown that high-quality exchanges can increase newcomers’ and leaders’ ability to adopt each other’s perspectives (Parker and Axtell, 2001; Ganegoda and Bordia, 2019). In doing so, newcomers are more likely to understand leaders’ expectations and execute tasks accordingly and effectively. More importantly, SET demonstrated that exchange goods are not limited to information (such as referent information about their own job responsibility), but also affect other elements (such as friendship; Homans, 1958; Omilion-Hodges et al., 2016). Frequent transactions and communications may generate a sense of mutuality and having an obligation to reciprocate. Previous research has shown that newcomers who perceive that they have high-quality LMX have a sense of relatedness and belonging beyond the employment contract (Ellis et al., 2019). All these feelings motivate the newcomers to work harder and feel obligated to repay leaders’ kindness (Sluss and Thompson, 2012; Ellis et al., 2019). Meta-analyses across cumulative studies demonstrated that LMX quality is positively related to job performance (Dulebohn et al., 2012; Martin et al., 2016).

Despite the increasing empirical evidence supporting the association between LMX and job performance cross-sectionally, it is still unclear whether changes in the quality of LMX are related to changes in newcomer performance. Previous research has demonstrated the dynamic nature of LMX in the first few months of the relationship (Nahrgang et al., 2009). Thus, LMX quality may fluctuate greatly in the early stages of organizational entry. The effects of such changes on newcomer performance are not known. According to SET (Cropanzano and Mitchell, 2005; Cropanzano et al., 2017), the benefits (consequences from the high-quality exchange) could generate self-enforcing cycles. Integrating this knowledge with the dynamic nature of newcomer socialization, we argue that when newcomers’ perceptions of the quality of LMX increase over time, newcomer performance is more likely to improve over time. With increasing LMX quality, newcomers may feel increased trust and support from leaders, which should generate increased feelings of obligation to reciprocate. Such obligation could encourage them to put more effort into work and thus translate to high job performance levels (Lee et al., 2019a). In contrast, if LMX quality decreases, it will be accompanied by decreased performance levels over time. Given the exchange of interpersonal relationships based on homeomorphic reciprocity (Cropanzano et al., 2017), if newcomers experience a decline in the quality of LMX, they may interpret this as an unfavorable exchange. Past research has demonstrated that poor interpersonal behavior results in detrimental behaviors (Andersson and Pearson, 1999; Lyons and Scott, 2012). More importantly, if leaders, as the formal authorities and direct supervisors, engage in poor interpersonal behavior, newcomers are more likely to be confused by this fluctuation with respect to the quality of LMX and start to feel uncertain about their jobs, thus leading to a decrease in their job performance. Therefore, we expect that the quality of LMX will be associated with newcomer performance both initially and as it changes over time.

***H1a:*** The quality of LMX is positively related to the initial level of newcomer performance.

***H1b:*** Increases in the quality of the LMX relationship are positively related to increases in the level of newcomer performance.

#### Moderator: Psychological Entitlement

Social exchange theory emphasizes that individual exchanges do not produce invariant reciprocity because individuals can vary in valuing the rule of reciprocity (Cropanzano and Mitchell, 2005; Love and Forret, 2008; Lee et al., 2019a). Whether or not a high-quality LMX promotes newcomer performance depends on the newcomers’ perspective on this relationship and the value they place on the rule of reciprocity. Psychological entitlement is an individual difference variable that captures the interest in power and inflated self-deserving minds. Psychological entitlement refers to an individual’s belief that they deserve more without consideration of actual contributions (Snow et al., 2001; Naumann et al., 2002; Harvey and Martinko, 2009; Lange et al., 2019). Psychological entitlement is a dominance-oriented personality trait (Lange et al., 2019). Highly entitled people have an inflated sense of their own power and prestige, so in a vertical relationship with the leaders, they may be less likely to consider themselves subordinates. Previous research has demonstrated that, compared to those with low psychological entitlement, employees with high psychological entitlement do not feel as high a level of obligation to their leaders even when LMX quality is perceived as higher than that of their coworkers (Lee et al., 2019a).

Highly entitled newcomers focused more on the achievements or the gains of all the workers. Because they feel more deserved than other people, they are inclined to pay more attention to the privileged status they think they deserve (Lee et al., 2019b) and less attention to actual job performance. More importantly, as LMX quality increases, leaders may provide more critical feedback to the followers to improve the performance. However, critical feedback may challenge newcomers’ positive self-image. Such a challenge may lead to disputes with their leaders and the inability to take feedback or advice seriously due to their self-serving mindset (Harvey and Martinko, 2009), which ultimately leads to poor performance.

Newcomers with a high level of psychological entitlement are more likely to lack the cognitive capacity to build an unbiased self-image (Harvey and Martinko, 2009; Harvey and Dasborough, 2015). This is also the reason that entitled individuals can maintain their inflated self-perceptions despite the objectively negative evidence. Therefore, when highly entitled newcomers experience a decrease in LMX, they are more likely to attribute this to their supervisors’ having a biased perspective. The research of Harvey et al. (2014) showed that highly entitled employees are more likely to perceive themselves as victims of abusive supervision. They may consider this to be abusive supervision, thus leading to a decrease in newcomer performance. Overall, we propose that psychological entitlement moderates the relationship between increases in LMX quality and the increases in newcomer performance.

***H2a:*** Psychological entitlement will attenuate the positive relationships between the initial quality of LMX and the initial level of newcomer performance.

***H2b:*** Psychological entitlement will attenuate the positive relationships between changes in the quality of LMX and changes in newcomer performance.

### CWX and Newcomer Adjustment

As highlighted in the previous section, leaders play a vital role during the newcomer socialization period. However, scholars found that newcomers’ relationships with coworkers play a more important role than our previously assumed (Takeuchi et al., 2011; Nifadkar and Bauer, 2016; Omilion-Hodges et al., 2016; Omilion-Hodges and Ackerman, 2018). Thus, in the present study, we also investigated the effect of CWX on newcomer adjustment.

Even though leaders have formal resources that only they can offer, coworkers are also key for newcomers’ adjustment to the organization and their role within it. With high quality CWX, newcomers may receive timely and referent information about their role in the organization and improve their task performance in a shorter time period. Past research on CWX showed that employees with high-quality CWX perform better due to their superior understanding of their role on the team (Chen et al., 2013). In team settings, many tasks require interdependence and rely on collaboration among team members (Wageman, 1995; Fang et al., 2017). Coworkers, as social models for newcomers, can provide newcomers with subtle and informal norms that leaders may not well understand, as they approach their team from a leader’s perspective (Wang et al., 2015). High-quality CWX facilitates the socialization process through an atmosphere of congenial teamwork and effective cooperation, which can be better leveraged to yield a more efficacious adjustment. For instance, the research of Banks et al. (2014) pointed out that high-quality CWX facilitated collaboration within a team. Korte (2010) also revealed that high-quality CWX forms a spirit of camaraderie among coworkers and helps newcomers work through difficult tasks or unmet negative expectations in work. Moreover, the support that newcomers obtain from high-quality CWX can facilitate the newcomer socialization process. High-quality CWX can provide both tangible (e.g., transmit information and resources) and intangible support (e.g., emotional support; Chiaburu and Harrison, 2008). For instance, Tews et al. (2013) pointed out that high-quality coworker relationships serve as a buffer to help newcomers deal with uncertainty.

In addition to the work environment, collaborations and exchanges between newcomers and their coworkers may boost the newcomers’ confidence at work. Being a new employee, newcomers should be eager to gain acceptance at work. Newcomers with high-quality CWX are more likely to feel accepted and empowered by their coworkers. Because newcomers are likely to work near or close to their coworkers daily, they need additional and more frequent assistance from and cooperation with their coworkers. They may be more eager to reciprocate their coworkers’ efforts both immediately and later on. In line with our theorizing above, we further propose that newcomers experiencing an increase in CWX quality see an improvement in job performance as well. For example, an increase in CWX quality provides newcomers with more organizational information (e.g., about the organization’s social, economic, and political environment) and a stronger foundation for collaboration and achieves an increase in newcomer performance. According to SET (Cropanzano and Mitchell, 2005; Cropanzano et al., 2017), once two parties start an exchange, positive patterns will be generated, and more rounds of reciprocation will occur. These reinforcing reciprocated rounds develop an atmosphere of congenial teamwork and facilitate collaboration. Furthermore, newcomers’ increases in CWX quality may lead to an increase in their positive affect, which may lead to deposits in a psychological capital bank. Previous research has demonstrated that an increase in psychological capital is positively related to job performance (Peterson et al., 2011). Therefore, we argue that not only that high-quality CWX has a positive effect on newcomer performance initially but also that this association continues over time.

***H3a:*** The quality of the CWX relationship is positively related to the initial level of newcomer performance.

***H3b:*** Increases in the quality of the CWX relationship are positively related to increases in the level of newcomer performance.

#### Moderator: Conscientiousness

Although newcomers can benefit from the horizontal exchange relationship with their coworkers, there are still certain newcomers who may not have the sense to learn from or make efforts to collaborate with their coworkers. Conscientiousness refers to the tendency to be reliable, responsible, and self-disciplined and to act according to one’s conscience (McCrae and John, 1992). Compared to less conscientious employees, highly conscientious employees present higher levels of emotional intelligence (Petrides and Furnham, 2001) and pay more attention to the relationship building (Roberts et al., 2009), which enhances trust and a friendly working atmosphere and facilitates team coordination. Therefore, we argue that a newcomer with high conscientiousness will be more likely to behave effectively at work when he or she has a better exchange relationship with his or her coworkers.

Highly conscientious people tend to devote more effort to perspective taking and appreciate the quality of relationships (Petrides and Furnham, 2001; Roberts et al., 2009). As time progresses, newcomers build higher-quality CWX relationships, and those who are high in conscientiousness are more likely to understand their coworkers’ points of view and take their coworkers’ advice, which facilitates the cooperation at work. Conversely, newcomers who are low in conscientiousness are less sensitive regarding the guidance and help from the coworkers (Greenhaus and Powell, 2006; Witt and Carlson, 2006). As they do not value the resources that coworkers provide, they are less likely to benefit from high-quality CWX. Moreover, newcomers with low conscientiousness and high-quality CWX may complain with their coworkers when they encounter obstacles instead of looking for professional help (Love and Forret, 2008). The research of Love and Forret (2008) showed that newcomers with high-quality CWX may not have a positive work attitude under troublesome circumstances. Hence, newcomers with low conscientiousness weaken the relationship between the quality of CWX and newcomer performance.

We further explore the potential moderation of conscientiousness in the relationship between the changes in CWX and the changes in newcomer performance. First, compared to less conscientious people, conscientious individuals tend to pay more attention to relationship goals, which focus on building high-quality relationships with others (Roberts et al., 2004, 2009). When newcomers experience an increase in CWX quality, they appreciate the comradeship formed with their coworkers. Thus, they devote more effort to accomplishing tasks more effectively and strengthening their comradeship. Second, conscientiousness pertains to a person’s integrity at work. Highly conscientiousness newcomers do not take the increased quality of their CWX for granted, and they repay their coworkers with highly efficacious group work. Therefore, conscientiousness is likely to impact the efficacy of CWX as a help-eliciting, reciprocal exchange process. Specifically, the beneficial effects of CWX on newcomers’ job performance are reciprocal-dependent. To the extent that highly conscientious newcomers have more alertness regarding the reciprocal exchange process, conscientiousness is likely to attenuate the hypothesized link between the increase in CWX and newcomer performance.

***H4a:*** Conscientiousness will strengthen the positive relationships between initial CWX quality and initial level of newcomer performance.

***H4b:*** Conscientiousness will strengthen the positive relationships between changes in CWX quality and changes in newcomer performance.

## Materials and Methods

### Sample and Procedures

We collected repeated measures from newcomers in high-tech firms. A random sample of 350 newcomers who had first been employed by the firm less than 6 months previously was selected for this research. All the newcomers worked on product development and improvement teams. HR representatives explained to the prospective participants that they would complete six waves of surveys at 6-week intervals. By adopting this longitudinal design, we were able to observe meaningful changes in newcomers’ LMX and CWX relationships and in their performance. Initially, their immediate supervisors were also contacted to assess the newcomers’ performance. The surveys were conducted online. At the end of each week, the participants received an email from the online system to alert them to complete the survey. The surveys were coded to allow us to match the participants across time. After the 6 weeks, we matched the six waves of data and skimmed them for possible abnormal response patterns. No such patterns were found.

At Time 1 (week 1), 350 newcomers were surveyed (we also asked their 70 immediate supervisors to assess their performance); 301 newcomers (representing a response rate of 86% of the full sample), and 61 supervisors completed surveys (representing a response rate of 87% of the full sample). At Time 2 (week 2), we sent the survey to all 301 newcomers who had previously responded and to their immediate supervisors. However, some of the newcomers had already left the original companies, and some of the supervisors did not respond. Thus, we obtained only 279 newcomer responses (80% of the full sample) and 56 supervisor responses (80% of the full sample). Then, in the 3rd week (Time 3), due to the aforementioned reasons, we received 261 completed responses from newcomers (75% of the full sample) and 53 supervisor responses (76% of the full sample). In the 4th week (Time 4), 249 newcomers and 50 supervisors filled out the surveys (71% of the full sample for newcomers; 71% of the full sample for supervisors). In the 5th week (Time 5), 237 newcomers and their supervisors participated (68% of the full sample). Finally, in the 6th week (Time 6), a total of 230 newcomer responses were received (66% of the full sample) and 46 supervisor responses were received (66% of the full sample). These high response rates were attributed to the encouragement provided by HR department and the salient value of our research topic (Roth and BeVier, 1998).

The average age of newcomer respondents was 25.56 years old (SD = 1.89). Forty percent of newcomer respondents were female. Additionally, 20.5% of newcomer respondents had diplomas (a 3-year high school), 49.5% of newcomer respondents had college degrees, and 30% had more advanced qualifications. Approximately 67% (66.8%) of the supervisor respondents were male; they averaged 37.8 years old (SD = 4.5). Additionally, 8.8% had diplomas, 56.2% of supervisor respondents had college degrees, with the remaining 34.0% holding more advanced qualifications.

### Measures

All measures that we used were originally developed in English. Although we collected our data in China, we strictly followed the translation and back-translation procedures of Brislin (1986).

#### Leader-Member Exchange Relationship

We used the 5-point Likert scale, LMX-7 scale developed by Graen and Uhl-Bien (1995). We asked the participants to describe to, during the past week, what kind of relationship they perceived that they had with their leader. A sample item was “During the past week, how would you characterize your working relationships with your leader?” Coefficient alpha for the scale was 0.88.

#### Co-worker Exchange Relationship

We adopt the LMX-7 measure (Graen and Uhl-Bien, 1995). The items were modified to reference one’s relationship quality with their coworkers instead of their supervisors. A sample item was “During the past week, how would you characterize your working relationship with your co-worker?” Coefficient alpha for the scale was 0.89.

#### Psychological Entitlement

Psychological entitlement was measured at the beginning of the data collection (at the Time 1) using the scale developed by Campbell et al. (2004). The scale contains nine items. The participants were asked to indicate the extent to which the items reflected their own beliefs. A sample item was “I demand the best because I’m worth it.” Coefficient alpha for the scale was 0.92.

#### Conscientiousness

Conscientiousness was measured at Time 1 using nine items taken from the Big Five scale from John and Srivastava (1999). The participants were asked to indicate the extent to which they agreed with the statement. A sample item was “I see myself as someone who is a reliable worker.” Coefficient alpha for the scale was 0.91.

#### Job Performance

We measured job performance by using the scale from Liden et al. (1993). We asked the participants’ immediate supervisor to assess his/her performance. The scale consisted of four items, including “Rate the overall level of performance that you observe for this member,” “This member is superior (so far) to other new subordinates that I’ve supervised before”; “What is your personal view of this member in terms of his or her overall effectiveness?”; “Overall, to what extent do you feel this member has been effectively fulfilling his or her roles and responsibilities?” Coefficient alpha for the scale was 0.83.

#### Control Variables

We controlled gender, age, and education.

### Analyses

We analyzed the data using a latent growth modeling (LGM) approach (McArdle, 2009) using R (lavaan package; R Core Team, 2015). The LGM approach is able to provide us the tool to directly observe the relationships among the changes of the variables. To access the fit of all models, we examined the chi square, the comparative fit index (CEI), the Tucker-Lewis index (TLI), the standardized root mean square residual (SRMR), and the root mean square of error of approximation (RMSEA). For the model fit, RMSEA ≤ 0.05 and SRMR < 0.08 represent an absolute model fit (Bollen, 1989; Browne and Cudeck, 1993; Marsh et al., 2004).

## Results

Table 1 presents descriptive statistics and intercorrelations for observed variables.

**Table 1:** Means, SDs, and Pearson correlation coefficients.

Variables	1	2	3	4	5	6	7	8	9	10	11	12	13	14	15	16	17	18	19	20	21	22	23
1. LMX1	-																						
2. LMX2	0.49	-																					
3. LMX3	0.35	0.42	-																				
4. LMX4	0.31	0.38	0.48	-																			
5. LMX5	0.33	0.38	0.37	0.42	-																		
6. LMX6	0.21	0.37	0.28	0.38	0.41	-																	
7. CWX1	−0.02	0.00	−0.08	−0.07	−0.10	−0.02	-																
8. CWX2	0.03	0.00	−0.06	−0.04	−0.04	0.07	0.47	-															
9. CWX3	−0.01	−0.03	−0.03	−0.06	−0.02	0.12	0.44	0.46	-														
10.CWX4	0.08	0.04	−0.07	0.03	−0.02	0.02	0.31	0.39	0.45	-													
11.CWX5	0.04	−0.03	−0.10	0.08	0.04	0.07	0.20	0.26	0.34	0.51	-												
12.CWX6	0.07	0.00	−0.06	0.05	0.02	0.07	0.26	0.35	0.34	0.44	0.51	-											
13.JOBP1	0.18	0.16	0.14	0.21	0.22	0.10	0.03	0.05	0.16	0.03	0.05	0.01	-										
14.JOBP2	0.16	0.19	0.20	0.09	0.19	0.13	0.00	0.09	0.04	−0.02	−0.02	0.07	0.47	-									
15.JOBP3	0.18	0.11	0.14	0.09	0.02	0.04	−0.01	0.03	0.00	−0.02	0.00	−0.04	0.37	0.56	-								
16.JOBP4	0.16	0.10	0.20	0.18	0.19	0.12	−0.02	0.08	0.10	0.01	0.01	0.04	0.39	0.26	0.43	-							
17.JOBP5	0.27	0.31	0.21	0.24	0.27	0.20	−0.07	0.05	0.07	0.01	0.08	0.11	0.38	0.27	0.23	0.45	-						
18.JOBP6	0.18	0.16	0.15	0.15	0.16	0.08	−0.01	0.07	0.14	0.06	0.17	0.22	0.26	0.25	0.21	0.35	0.49	-					
19.Pentitle	0.16	0.04	0.02	0.01	0.07	−0.01	−0.10	0.03	−0.03	−0.01	−0.01	−0.08	0.06	0.06	0.09	0.13	0.20	0.10	-				
20.Conscient	−0.02	0.03	−0.02	0.04	−0.01	−0.04	−0.03	0.04	−0.01	0.13	−0.03	0.00	−0.01	−0.03	0.06	0.00	0.04	0.03	0.28	-			
21.Gender	0.02	0.07	0.03	0.02	0.12	−0.06	0.07	0.02	−0.04	−0.05	0.02	0.00	−0.07	−0.03	0.00	−0.06	0.02	0.09	−0.05	−0.02	-		
22.Age	0.05	0.13	0.02	−0.13	−0.03	−0.05	0.03	0.00	0.03	0.03	0.01	−0.03	−0.02	0.01	0.01	0.03	−0.04	−0.05	−0.03	−0.03	0.02	-	
23.Education	0.01	0.14	0.06	0.04	0.01	0.10	0.08	0.03	0.15	0.02	0.06	0.07	0.09	0.00	0.00	0.03	0.09	−0.13	−0.06	−0.06	0.09	0.04	-
M	4.69	4.66	4.66	4.62	4.60	4.62	4.62	4.62	4.66	4.66	4.65	4.63	4.53	4.59	4.59	4.57	4.55	4.53	4.56	4.61	0.40	25.56	2.10
S.D.	0.51	0.57	0.56	0.55	0.56	0.54	0.54	0.55	0.57	0.56	0.53	0.57	0.59	0.62	0.61	0.62	0.61	0.59	0.58	0.56	0.49	1.90	0.70

N = 230. For all correlations above |0.12|, *p* < 0.05. LMX, leader member exchange; CWX, coworker exchange; JOBP, job performance; Pentitle, psychological entitlement. 1 = Time1; 2 = Time 2; 3 = Time 3; 4 = Time4; 5 = Time 5; 6 = Time 6.

First, we examined whether focal study measures differ from each other at each of the six measurement occasions by a series of confirmatory factory analyses. The results, presented in Table 2, indicated that the hypothesized three-factor model (LMX, CWX, and job performance) provided an adequate fit to the data at each of the measurement waves (T1–T6):*χ*^2^[132] ranges from 123.37 to 153.19, goodness-of-fit (CFI) ranges from 0.99 to 1.00, TLI ranges from 0.99 to 1.00, RMSEA ranges from 0.00 to 0.03. The evidence shows the repeated measures were distinct from each other over the six measurement occasions.

**Table 2:** Model fit statistics for testing discriminant validities and measurement invariance.

Model/variable	*χ*^2^	*df*	CFI	TLI	SRMR	RMSEA	△*χ*^2^	△*df*
Measurement model (three factors: LMX, CWX, and JOBP)								
Measurement model T1	124.84	132	1.00	1.00	0.04	0.00		
Measurement model T2	146.47	132	0.99	0.99	0.04	0.02		
Measurement model T3	146.96	132	0.99	0.99	0.05	0.02		
Measurement model T4	153.19	132	0.99	0.99	0.05	0.03		
Measurement model T5	143.36	132	0.99	0.99	0.05	0.02		
Measurement model T6	123.37	132	1.00	1.00	0.04	0.00		
Longitudinal measurement invariance across four waves								
Configural invariance CFA	838.19	792	0.99	0.99	0.04	0.02		
Metric invariance CFA	910.18	867	0.99	0.99	0.05	0.02	71.84	75

LMX, leader member exchange; CWX, coworker exchange; JOBP, job performance.

Next, due to the measurement invariance as a prerequisite for subsequent analysis using LGM (Chan, 1998), we examined the measurement invariance of repeated measures (i.e., LMX, CWX, and job performance) over the six measurement occasions. The measurement invariance results, presented in Table 2, indicated that each configural invariance model received reasonable fit to the data. More importantly, the addition of metric invariance constraints in each metric invariance model did not result in significantly worse fit to the data, supporting the metric invariance of the current measurement. Therefore, the measurement invariance tests satisfied the assumption for conducting LGM.

**Figure 1:**
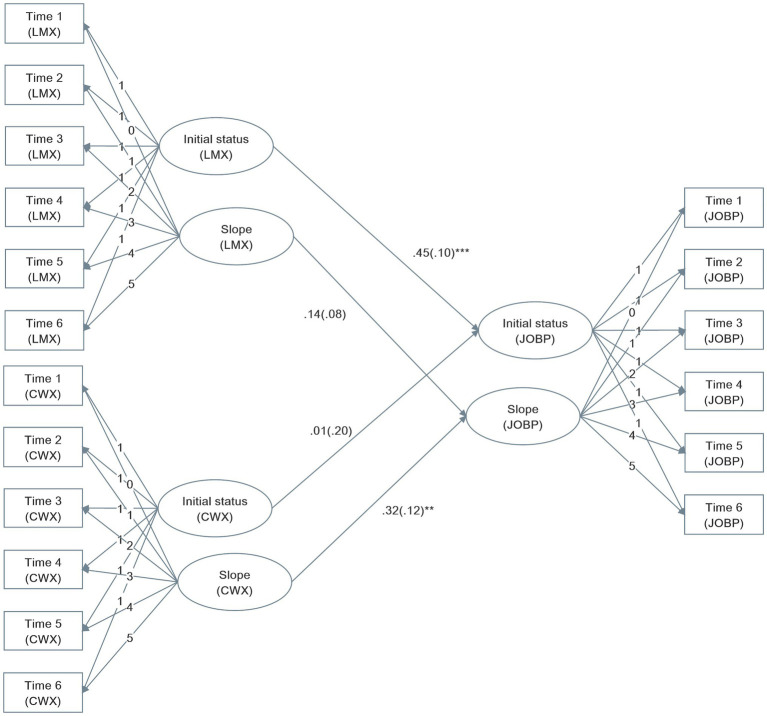
Parameter estimates in the testing model. LMX, leader member exchange; CWX, coworker exchange; JOBP, job performance.

Hypothesis 1 predicts that (a) the initial status of and (b) increases in LMX are positively related to the initial status of and increases in newcomers’ job performance over time, respectively. As shown in Figure 1, the initial factor of LMX is indeed positively and significantly related to the initial factor of job performance (*β* = 0.45, *p* < 0.001), supporting Hypothesis 1a. However, inconsistent with our expectation, the slope factor of LMX did not significantly related to the slope factor of job performance (*β* = 0.14, n.s.).

Hypothesis 2 states that psychological entitlement will moderate the relationship between the initial status of /increases in LMX and the initial status of/increases in newcomers’ job performance. Partially supporting Hypothesis 2, the results, presented in Table 3, indicated that the relationship between the initial status of LMX and the initial status of job performance is significantly stronger (Δ*β* = 0.37, *p* < 0.05) when psychological entitlement is lower (*β* = 0.58, *p* < 0.001) rather than it is higher (*β* = 0.22, n.s.). However, the results revealed that psychological entitlement did not moderate the link between the slope factor of LMX and the slope factor of job performance.

**Table 3:** Results of structural model analysis for high and low task uncertainty groups.

Moderator	LMX_initial_ to JOBP_initial_	LMX_slope_ to JOBP_slope_	CWX_initial_ to JOBP_initial_	CWX_slope_ to JOBP_slope_	△*χ*^2^	△*df*
Psychological entitlement					4.61^*^	1
Low	0.58(0.12)^***^	0.21(0.13)	−0.17(0.55)	0.36(0.16)^*^		
High	0.22(0.13)	0.03(0.10)	0.11(0.19)	0.31(0.20)		
Conscientiousness					5.12	1
Low	0.41(0.15)^**^	0.07(12)	0.02(0.27)	0.07(0.16)		
High	0.50(0.12)^***^	0.21(0.11)^*^	0.12(0.24)	0.57(0.17)^**^		

* *p* < 0.05;

** *p* < 0.01;

*** *p* < 0.001.

Hypothesis 3 predicts that (a) the initial status of and (b) increases in CWX are positively related to the initial status of and increases in newcomers’ job performance over time, respectively. As shown in Figure 1, the slope factor of CWX is indeed positively and significantly related to the slope factor of job performance (*β* = 0.32, *p* < 0.01), supporting Hypothesis 2b. However, inconsistent with our expectation, the initial status of one’s CWX did not significantly related to the initial status of one’s job performance (*β* = 0.01, n.s.).

Hypothesis 4 states that conscientiousness will moderate the relationship between the initial status of /increases in CWX and the initial status of/increases in newcomers’ job performance. Partially supporting Hypothesis 4, the results, presented in Table 3, indicated that the relationship between the slope factor of CWX and the slope factor of job performance is significantly stronger (Δ*β* = 0.50, *p* < 0.05) when one’s conscientiousness is higher (*β* = 0.57, *p* < 0.01) rather than it is lower (*β* = 0.07, n.s.). However, the results revealed that one’s conscientiousness did not moderate the link between the initial status of CWX and the initial status of job performance.

## Discussion

This study aims to investigate whether newcomers’ interpersonal relationships, during the socialization period are linked to their initial performance and their performance over time. More specially, we investigated whether high-quality LMX and CWX lead to better newcomer performance and whether newcomer performance improves over time with greater increases in the quality of LMX and CWX. The results of this study showed that initially, newcomers’ LMX quality is positively related to newcomer performance, but an increase in LMX quality was not significantly related to an increase in newcomer performance; additionally, newcomers’ CWX quality was not related to their performance, but changes in the CWX quality were related to improvement in newcomer performance over time.

### Theoretical Implications

By investigating the relative effect between LMX and CWX, our study highlighted the important role of coworkers in the socialization period. Many scholars studying interpersonal exchange relationships have argued that newcomers’ interaction and exchange with their leaders are central to newcomer adjustment at work (Jokisaari and Nurmi, 2009). Our study extends earlier research by showing that coworkers also play a role in newcomer socialization. Future research may take our findings into consideration when drawing conclusions about the implications of exchange relationships for newcomer socialization. Previous research has shown that the quality of newcomers’ exchange relationships with the organizational insiders (e.g., leaders and coworkers) benefits newcomer performance (Tse and Dasborough, 2008), but our results show that two exchange relationships (i.e., LMX and CWX) facilitate newcomer socialization and do so in different ways. Support from supervisors is indeed most crucial at the beginning, and collaboration with coworkers becomes more essential thereafter. Thus, scholars should not simply focus on LMX, but also consider that the development of CWX quality can also greatly benefit for newcomer performance.

Moreover, our results show that there is no significant association between changes in the quality of LMX and changes in newcomer performance but that changes in the quality of CWX were significantly related to changes in newcomer performance. One possible explanation is that changes in the quality of LMX, whether it increases or decreases, may confuse newcomers, making the newcomers uncertain about their work role. Such confusion causes the unstable performance, and cannot generate a systemic change pattern. However, newcomers become more familiar with their coworkers than their superiors in work settings, so they are more likely to make sense of coworkers’ behavior changes (Louis, 1980; Chen et al., 2011). Thus, an in-depth study is needed to further explore the underlying mechanism of the beneficial effect of these exchange relationships.

Our study also adds to earlier research on socialization by demonstrating how changes in the exchange relationship over time are related to changes in the key outcome of newcomer adjustment. Moreover, our study contributes to the literature by indicating that it may be appropriate to examine the initial level of and the changes in these relationships simultaneously. In terms of the link between LMX and CWX and performance, studies have often been concerned with whether newcomers have a good or bad relationship with their leaders; thus, it is particularly important for researchers to adopt a dynamic perspective that repeatedly tracks newcomers’ LMX quality. This is because simply observing that a high level of LMX quality across newcomers is associated with a high level of performance is not direct evidence of increased performance or the positive trajectory of performance. Moreover, our research provides empirical evidence that increases in a newcomer’s CWX quality over time, rather than in his or her initial CWX quality, positively predict his or her performance, demonstrating the importance of examining changes in CWX. Studies have often been interested in whether newcomers have perceived better or worse relationships with their leaders. However, past research neglected the resources that may be held by coworkers, given that they may have more experience with the tasks of newcomers than the latter do themselves. Moreover, our findings revealed a positive effect of change in CWX quality on change in newcomer performance, suggesting that newcomers who perceive increases in CWX quality experience an improvement in performance. This change-to-change effect extends prior research that has demonstrated a level-to-level effect of CWX on newcomers’ behaviors (Nifadkar and Bauer, 2016). Our study findings are also consistent with the broader literature on newcomer performance, which states that researchers should not assume that the variables predicting the initial level of newcomer performance would also influence changes in newcomer performance (Ployhart and Hakel, 1998; Chen, 2005). Future research should, therefore, adopt a more dynamic perspective and use longitudinal designs to explore both intraindividual changes and interindividual differences in LMX/CWX and performance.

Our research also contributes to the LMX and CWX literature by identifying psychological entitlement and conscientiousness as novel boundary conditions. According to Blau (1964), every individual values the reciprocation differently. Perugini et al. (2003) demonstrated that people’s responses to social exchange appear to vary signficantly. The positive effect of LMX/CWX on newcomer performance may depend on the degree to which a newcomer values the rule of reciprocation. This study provides support for Blau and Perugini et al.’s claim. Consistent with prior research (Lee et al., 2019b), psychological entitlement inhibits the vertical relationship between the quality of LMX and employee outcomes. We rationalize that this finding arises from self-inflated views on leaders’ empowerment, which fit newcomers’ inflated sense of their deservingness in this vertical relationship. Our findings also advance the horizontal exchange relationship (i.e., CWX) by identifying conscientiousness as a boundary condition that determines the extent to which the quality of CWX change impacts newcomer performance change. In line with prior conscientiousness research that views conscientiousness as a good quality, we found that the positive relationship between the change in the quality of CWX and change in newcomer performance is stronger for newcomers with high conscientiousness than for those with low conscientiousness. Our results show that employees with high conscientiousness are likely to sense the changes in these relationships and respond with better or worse performance. A newcomer’s sense of the necessity to reciprocate and build a good working environment could influence the exchange relationship development process. An increase in the quality of CWX may not always explain how newcomer performance changes. Rather, conscientiousness is a necessary precondition for linking changes in the quality of CWX to newcomer performance.

### Practical Implications

Our findings have implications for future managerial practice. First, managers should encourage the development of a helping culture and build a friendly working environment. As our results suggested, newcomers’ interpersonal relationships with their leaders and coworkers may affect their job performance in different ways. For employees starting a job in a new organization, quality interpersonal relationship building need not be limited to conventional LMX. Rather, the long-term CWX development may be leveraged to enhance their performance in the long run. Second, although managers should empower newcomers, they should also consider the personality of newcomers. Excessively high-quality LMX may lead highly entitled newcomers to focus less on their job and to mistake their empowerment for entitlement. Third, the effect of a good-quality relationship on job performance depends upon the level of the newcomer’s conscientiousness. As shown in our findings, in the long term, conscientiousness newcomers may repay good quality with better long-term performance. In summary, our research provides insights into interpersonal relationships with short- and long-term views and encourages managers and newcomers to value both good-quality of LMX and good-quality CWX.

### Limitations

Our research has several limitations. First, our study is limited in that we examine only the relationship of LMX/CWX to newcomer performance. In addition to the dispositional factors (e.g., psychological entitlement and conscientiousness) in our study, there may still be other situational (e.g., leadership style) or other dispositional factors (e.g., self-efficacy) that impact newcomer performance. Future research is needed to further uncover the boundary conditions of the positive linkage between LMX/CWX and performance. Second, given our focus on newcomer adjustment, we only examined newcomer in-role performance. Prior research has already demonstrated that organizational citizenship behavior is a crucial outcome of LMX and CWX (Götz et al., 2020). Future research should also pay attention to the newcomers’ out-role performance and out-role performance improvement. Moreover, we also encourage future scholars to explore other proximal outcomes such as organizational commitment. Past research indicated that the increased newcomers’ feedback-seeking behavior with their leaders formed a strong exchange relationship, and contributed to increases in organizational commitment (Vandenberghe et al., 2021). Third, our findings may have limited generalizability because we collected our research data from China. Although our framework did not involve any contextual factors, we still cannot neglect the influence of national cultures. Chinese relationship philosophy in particular may have had some systemic influence on our data. Thus, we encourage the future research to examine our research questions in other countries. Fourth, although we observed the changes in LMX/CWX and performance, we did not examine different forms of change (e.g., nonlinear change trajectories). Future research should consider the different forms of change to further uncover the dynamic relationships between newcomers and their leaders and coworkers.

## Data Availability Statement

The raw data supporting the conclusions of this article will be made available by the authors, without undue reservation.

## Ethics Statement

Ethical review and approval was not required for the study on human participants in accordance with the local legislation and institutional requirements. Written informed consent for participation was not required for this study in accordance with the national legislation and the institutional requirements.

## Author Contributions

C-RL and JL conceived the project and designed the study. JL drafted the manuscript, conducted many revisions, and oversaw the whole research process. C-RL collected and analyzed the data. AL provided the guidance, commented on the drafts, and conducted many revisions. XL provided the guidance and commented on the final draft. All authors contributed to the article and approved the submitted version.

### Conflict of Interest

The authors declare that the research was conducted in the absence of any commercial or financial relationships that could be construed as a potential conflict of interest.
